# Medical Opinion and Movement

**Published:** 1908-10-10

**Authors:** 


### Medical Opinion and Movement.

Myasthenia gravis forms the subject of an extensive article in *La Semaine Médicale* by Professor G. Marinesco, of Bucharest. He surveys the research work of others and the various hypotheses held concerning the pathology of this disease, and at the same time he submits a report of his own observations upon two cases of the disease occurring in sisters. This last fact is in itself remarkable, as there appears to be no other observation on record suggesting a family tendency for the disease. Sir Samuel Wilks first observed the clinical symptoms of the disease in 1877, but Erb studied it more closely and defined it quite clearly as characterised by a paresis, accompanied by fatigue and exhaustion of the muscles under the control of the cranial motor nerves, extending often to the other skeletal muscles of the body. He supposed the underlying pathological lesion to be a polio-encephalitis. Many cases, however, occurred which failed to show post-mortem any anatomical lesion. Then in 1901 Weigert reported a case of the disease in which there was a malignant tumour in the thyroid region with the characteristic structure of the thymus, and, furthermore, he observed infiltration of the muscles by cells identical with those of the growth. Many cases have since been reported in which lymphoid and other changes in the muscles are observed, together with abnormal conditions of the thymus gland. In others fatty infiltration and degeneration of the muscle fibres are described, and the question arises whether these changes are the primary lesion of the disease, or whether they are secondary to some intoxication, probably of the nature of a disturbance in the normal internal secretions of the body.

Professor Marinesco's cases are typical instances of the disease, and they developed in the characteristic manner. One of them succumbed to pulmonary phthisis, and post-mortem examination revealed an extensive fatty infiltration of the muscles, with multiplication of the nuclei of the sarcolemma, the changes in the several muscles being generally proportional to the paresis present during life. In addition he notes an enlargement of the hypophysis and certain microscopic changes in this organ, and also in the parathyroids, thymus, thyroid, and suprarenals. The author, in his interesting discussion of the pathology of the disease, lays emphasis upon muscular fatigue as the peculiar symptom of the disease, and regards this as the key to the elucidation of its pathology. He carried out a series of experiments on his patients with the ergograph and dynamometer, which clearly demonstrated the phenomena of fatigue so characteristic of the disease. Moreover he claims to be able to produce the same myasthenic reactions of fatigue to stimulation, both electrical and voluntary, in a normal individual by causing ischæmia or hyperæmia in a limb by means of an Esmarch's bandage. From these facts he attributes those fatigue symptoms to diminished power of oxidation either by a failure in the supply of the supposed oxidizing enzymes of the body or by the production of some toxic substance which inhibits the normal oxidation process in the muscles. In consequence the products of muscular activity accumulate, and failing to be oxidised in the normal way to carbonic acid and water, have a toxic effect upon the muscles and cause the signs of fatigue. Kaufmann has shown by careful examination of the excreta of a myasthenic that among other changes in the urine during muscular activity the proportion of nitrogen excreted in the urine is enormously increased, suggesting a destruction of albumin. According to this author, therefore, the underlying pathology of this disease is chemical in nature, consisting in a deficient power of oxidation, determined by some abnormal condition of the glands of internal secretion, and resulting in the clinical symptoms of muscular fatigue and paresis, and the anatomical changes in the striated muscles.

Bier's hyperæmic method of treatment will soon appear to approach very closely to a universal panacea for all the ills of the flesh. Fresh fields of usefulness are being discovered almost daily for its application. One of its latest triumphs appears to be its efficacy in the treatment of ununited fractures. Dr. Gangitan reports the successful treatment of ununited fractures of the leg in three instances. The patients were peasants somewhat advanced in years, the ages being between fifty and seventy, and in each case, after the limb had been in splints for periods of three to five months, the bones remained still ununited. An elastic bandage was applied to the limb high up in the thigh for an hour night and morning, care being taken that the limb was warm and free from pain. After the application in the morning the patient was kept in bed for a time and then allowed to get up and walk about with crutches. No massage was used. At the end of a month complete union had occurred, and the patients were able to get about, in spite of the fact that they had been bedridden for several months previously. Unfortunately the method would be difficult to apply for certain cases of fracture, in which union is specially difficult to secure—namely, intracapsular fracture of the femur and fractures in the neighbourhood of the acromion process of the scapula.

It may be remembered that some two years ago Bordet and Gengou succeeded in isolating from the expectoration of patients suffering from whooping-cough a small micro-organism, ovoid in shape, which they regarded as the specific microbic agent of the disease. Dr. Klimenko, a Russian physician, has recently reported upon his own research work, which confirms so far the work of these previous authors. In the first place he was able to obtain from the expectoration of five children in the early stage of the disease pure cultures of the bacillus described by Bordet and Gengou. Injection of these cultures into guinea-pigs and rabbits gave negative results, but a sucking-pig and a lamb similarly treated developed fever with diarrhœa lasting three or four days. He obtained, however, much more definite results with seven monkeys. Five were inoculated artificially and two contracted the disease by infection from the others. In view of the fact that the disease has been occasionally observed in dogs, three adult dogs and forty-eight puppies were inoculated. The adult dogs showed immunity, but the puppies developed the disease with all its characteristic symptoms, corresponding in all respects to the clinical course of the disease in children. Moreover, the author claims to have obtained from the pneumonic patches of an infant who succumbed to the disease a bacillus identical with that found in similar pneumonic patches of the puppies inoculated with the bacillus above mentioned.

---

THE first case of Asiatic cholera was reported in St. Petersburg on August 22 last. Its advent was by no means unexpected: ever since the outbreak of the disease in Southern Russia the authorities had been making strenuous and valiant efforts to improve the sanitary conditions of the capital. That this was a difficult—and in many ways a thankless—task can be seen from the official reports published in the Russian professional papers; but the State Board of Health in Russia is excellently controlled, has far-reaching powers, and appears to be unhampered by red tape. The fight against the cholera in the capital was commenced by a meeting of the Sanitary Committees jointly. At this meeting very drastic regulations were drawn up, which received the sanction of the ministry, and which, it is satisfactory to know, have been rigorously enforced since. Thus second-hand dealers were forbidden to buy or sell clothes except under strict supervision; no second-hand clothing was to be exposed for sale except when disinfected by the sanitary officials. Similar restrictions were made with regard to second-hand furniture dealers. The city was divided into fourteen sanitary sectional districts, each patrolled night and day by a specially trained sanitary staff. To help these workers, flying sanitary columns, the members of which are paid by the central board, have recently been organised. Inoculation depots have been established at which persons who desire to be inoculated are treated free of charge, but such inoculation has not been made compulsory. All medical students, male or female, who are working as volunteer assistants under the sanitary committees, are excused attendance at their lectures and practical classes. The result of this energetic action on the part of the authorities has been extremely satisfactory, and although the sanitary condition of the capital was by no means what one could have desired, the disease has been very effectively coped with. The latest statistics show that the percentage of deaths to population is smaller in St. Petersburg than anywhere else in Russia. Curiously enough, it is very low in the Don Cossack districts, although the percentage of cholera patients is far higher there than in other districts. The highest mortality figures are furnished by the districts of Astrachan, Kuban, and Nijni-Novgorod. At present it is too soon to draw conclusions from the interesting returns furnished by the official reports, but the latter are so full and detailed that they promise to prove very valuable for future reference.

---

"ZAMBESI ULCER" is a condition which is familiar enough to most students of tropical diseases, though hitherto it has scarcely received the attention it deserves. During the late South African war it was frequently brought to the notice of military surgeons under its more common name of "veld sore," and at the time attracted some pathologists, who endeavoured to group it with the so-called phagedænic ulcers. Its causation and its pathology are, however, much disputed. Dr. Ashley-Emile's paper in the *South Africa Medical Record*, however, throws a new ray of light upon the matter. He announces the discovery of the larvæ of a dipterus fly in the recesses of the ulcer in one of his patients, and concludes that the lesion is due to direct infection with these larvæ, which cling to the long "Zambesi grass," either directly, as when the native walks through the grass or indirectly by grubs being brought back in the clothes and are deposited in the huts. The larvæ soon burrow beneath the naked skin, and cause a rapid local necrosis, usually preceded by a vesicular stage. Untreated cases may result in extensive sloughing; the underlying muscles are attacked, and even the periosteum and bone. As the extensor surfaces of the legs are more often affected than the flexor surfaces, curious deformities may result, the most frequent being "a kind of talipes equinus." Clinically the ulcer, with its sharply cut, indurated edges, and dusky red areola, is apt to be mistaken for a specific lesion. By removing the central slough however, characteristic white points, the papillæ, are found. In treating such cases the author finds extraction of the larvæ gives the best results. When the superficial fascia has been pierced, however, extraction is a matter of great difficulty, and the practitioner has to be content with vigorously scraping the ulcer and the employment of germicides.

---

CRANWELL contributes to the *Semana Medica* an interesting study of the diagnosis and treatment of diaphragmatic hernia based on a series of original observations. After carefully tabulating the recorded cases of the lesion, he finds that the viscus most often involved is the stomach, followed, as is to be expected, by the colon, large intestine, and omentum. Diagnostically his cases presented no out-of-the-way features, as in all there was a marked history of injury. A point which is useful for future reference is that such lesions may often be helpfully screened. The radiogram shows the protrusion into the thorax, and clearly establishes the diagnosis in doubtful cases where the symptoms of intestinal obstruction are apt to lead the medical attendant astray. Clinically diaphragmatic hernia is a rare lesion, though interesting cases, most of them of congenital origin, are from time to time reported. The diagnosis is often very difficult, in many cases impossible, to establish with certainty; but where there is evidence of old injury to the diaphragm—such as an old stab or bullet wound—the probability of the case being one of diaphragmatic rupture must never be lost sight of. Very little help is gained from auscultation or bimanual examination. Usually the symptoms of internal strangulation outweigh all others, and the case is diagnosed, rightly enough, as one of obstruction. The only treatment is immediate operation. The author lays stress on the importance of attempting a radical cure in all chronic cases, and prefers the transpleural route for the operation.

---

SOME recent experiments of Sticker (*Berl. Klin. Woch.*) with regard to the treatment of malignant tumours by atoxyl have shown that this drug by itself is incapable of producing any marked improvement in the condition of the patient or any marked retardation of the growth of the tumour. The experiments were for the most part carried out upon dogs, the subjects of sarcomatous growths. The toxic effects of the accumulated drug were such as to produce great disturbance of the health of the animals. Sticker next tried the effect on the tumours of injecting locally the blood of other animals, and found the blood of a ram was the most active, although the results were not permanent. A repetition of this treatment greatly upset the general health of the animal. On combining the atoxyl treatment with local injections of ram's blood, however, the author found that the toxicity of both therapeutic agents was markedly diminished and their influence on the growths markedly increased. Next trying his new treatment on the human subject, injections of dog's or ram's blood (1.5 to 4.5 c.c.) caused cutaneous epitheliomata to shed their cuticle with great rapidity. Although they cannot be said to justify the belief that injections of atoxyl combined with heterogeneous albumins are a certain cure for cancerous growths, the author's results are of great interest, and would seem to justify the hope that cancer is, after all, a curable disease.

---

IT is sometimes necessary to perform an operation upon a patient who is not a suitable subject for a general anæsthetic. Local anæsthesia procured by the subcutaneous injection of cocaine, novocaine, or some similar drug, is not always of sufficient degree when the operation contemplated is extensive. It has struck Bier that a combination of his method of procuring venous stasis and the intravenous injection of a solution of novocaine into a vein lying between the ligatures might meet the difficulty. At a recent meeting of the German Surgical Society in Berlin, he communicated the results of two cases so treated. In the first case, that of a patient suffering from advanced tuberculous disease of the elbow, ligatures were applied above and below the joint, and an injection of novocaine given into the median basilic vein between the ligatures. Complete anæsthesia was thereby obtained. In the second case, that of an amputation through the thigh, a similar procedure was equally successful. From these two cases Bier is led to believe that a sufficient degree of analgesia is almost always obtained if the procedure be carefully carried out. He recommends that the vein chosen for the injection should be a subcutaneous one, *e.g.* one of the saphenous veins in the leg, or the median basilic or cephalic in the arm. The method is particularly useful in amputations. The strength of novocaine used depends on the locality of the site of operation. In the arm 100 cc. of a ¼ per cent. solution usually suffices; in the leg 150 cc. of a ½ per cent. solution is more useful. Novocaine is always soluble in a saline solution, its use is attended with practically no danger, and the anæsthesia passes off almost as soon as the elastic ligatures are removed.

---

ALTHOUGH it is but rarely that a complete cure of tabes by anti-syphilitic remedies can be cited, yet there are numerous cases on record in which the disease has been definitely arrested in its early stages by this form of treatment. Dr. Milian in *Le Progrès Médical*, gives his experiences, extending over a period of eight years, in the treatment of ten cases of the disease, in all stages. The cases have been regularly treated with anti-syphilitic drugs. In no case has the disease progressed, or new symptoms developed, and in some cases old symptoms have gradually passed away. Only one patient has shown no improvement, and in this case mercurial injections could not be tolerated on account of digestive and nervous troubles which immediately followed the treatment. The author is led to think that there is no truth whatever in the theory held by some that mercury is the cause of the tabetic condition. It is true that in the course of treatment of tabes by mercurial injections, lightning pains in some cases only manifest themselves after the injections, or in other cases are intensified by the treatment, but usually the mercury acts as a sedative to these pains, especially in advanced tabes.

---

BAGINSKY, in the *Berlin. Klin. Woch.*, has gathered together the statistics of an epidemic of diphtheria which occurred in Berlin in 1907. He divides the cases into slight, severe, and very severe. In the first group the percentage was 23.2, in the second 44.4, and in the third 32.3. It will thus be seen that the epidemic was a somewhat severe one. The mortality was 11.9 per cent. of cases treated, which includes death from complications, such as tubercle, &c. Antitoxin was given as a routine practice, but rarely in greater doses than 5,000 units. The author is of opinion that higher doses than this are of no use in preventing subsequent cardiac troubles or paralyses. He is also convinced that it will never be possible entirely to prevent mortality from the disease by the use of serum, no matter how skilful be the technique employed. He admits, however, that there is still room for improvement in the results obtained by serum treatment. He thinks that the treatment is still often delayed too long, and not employed in a rational manner.

